# Neighborhoods, Schools and Obesity: The Potential for Place-Based Approaches to Reduce Childhood Obesity

**DOI:** 10.1371/journal.pone.0157479

**Published:** 2016-06-16

**Authors:** Brian Elbel, Sean P. Corcoran, Amy Ellen Schwartz

**Affiliations:** 1 New York University School of Medicine, Department of Population Health, New York, NY, United States of America; 2 Robert F. Wagner Graduate School of Public Service, New York, NY, United States of America; 3 New York University Steinhardt School of Culture, Education and Human Development, New York, NY, United States of America; 4 Maxwell School, Syracuse University, Syracuse, NY, United States of America; Philipps University Marburg, GERMANY

## Abstract

A common policy approach to reducing childhood obesity aims to shape the environment in which children spend most of their time: neighborhoods and schools. This paper uses richly detailed data on the body mass index (BMI) of all New York City public school students in grades K-8 to assess the potential for place-based approaches to reduce child obesity. We document variation in the prevalence of obesity across NYC public schools and census tracts, and then estimate the extent to which this variation can be explained by differences in individual-level predictors (such as race and household income). Both unadjusted and adjusted variability across neighborhoods and schools suggest place-based policies have the potential to meaningfully reduce child obesity, but under most realistic scenarios the improvement would be modest.

## Introduction

Childhood obesity is a pressing public health and policy concern in the United States. Nationally, nearly seventeen percent of children are obese [1], and even more are overweight and at high risk of obesity later in life. Moreover, there have been no significant changes in obesity prevalence in youth between 2004 and 2012 [1]. The consequences for children are substantial, with long-term negative impacts on health and life chances (e.g., income, employment) [2] and potential short-term impacts on fitness, social and emotional development (at least partially driven by obesity-related stigma), and academic success [3, 4]. That the prevalence of child obesity is unevenly distributed across sociodemographic groups is of special concern, with disproportionate numbers of racial/ethnic minorities and low-income youth obese or overweight [5–8]. Despite these well-documented disparities, we have limited evidence on the extent to which these gaps are explained by individual characteristics, behaviors, and family resources, or differences in the environments in which children spend most of their time: neighborhoods and schools.

The role of individual versus environmental risk factors are of particular interest to policymakers weighing *place-based* approaches to combating childhood obesity [9]. These include policies targeted to specific geographic locations, including neighborhoods and schools [10]. They also include policies to expand access to affordable, healthy food, and locations for regular physical activity. For example, efforts to bring full-service grocery stores and fresh produce to so-called “food deserts”—low-income neighborhoods lacking access to supermarkets or other fresh food sources [11]—are often cited as useful strategies [12–14]. Recent federal and state legislation to improve the nutritional quality of school meals and remove competitive, less healthy, foods from schools are another example [15, 16]. Strategies to improve neighborhood walkability, increase access to parks and open spaces for physical activity, and raise physical education requirements in schools are others [17].

While there is growing empirical evidence on specific interventions and policies to reduce the prevalence of obesity (as noted above), there remains a paucity of research documenting the overall, relative contribution of neighborhoods and schools versus sociodemographic risk factors (like race, income, immigration status) to obesity, particularly for urban schoolchildren. This is, in part, due to limitations in available data. Nationally representative datasets such as the National Health and Nutrition Examination Survey (NHANES) include requisite information on obesity and family background but have relatively small samples of students in any one city and rarely more than a handful in any one school or neighborhood, making it difficult to parse the effects of schools and neighborhoods. Longitudinal data on child obesity is even rarer.

This study uses richly detailed data on the body mass index (BMI) of nearly all New York City public school students in kindergarten through 8^th^ grade to document variability in obesity across NYC neighborhoods and schools, and estimate the extent to which this variation can be explained by individual-level predictors such as race and household income. In 2005–06, the NYC Department of Education (NYCDOE) adopted the *Fitnessgram*, an annual measurement of the height, weight, and physical fitness of almost every student [18]. By 2012–13, the *Fitnessgram* covered roughly 875,000 students in 1,650 schools citywide. We combined these data with other information from the NYCDOE, including student race/ethnicity, age, gender, recent immigrant status, income eligibility for subsidized meals, and home address, to examine how the prevalence of obesity varies across neighborhoods and schools, both before and after adjusting for these observed student characteristics.

Together, these unadjusted and adjusted measures of variability across neighborhoods and schools are used to assess the potential for place-based approaches to reduce child obesity. For instance, evidence of significant spatial variation in the prevalence of obesity would reveal opportunities to target interventions to neighborhoods or schools where rates of obesity are the highest. These measures could further be used to identify context-level factors systematically associated with obesity [19]. Alternatively, if much of the variation in obesity across neighborhoods and schools is explained by differences in student populations, this would suggest that the focus of interventions should be on populations, not necessarily place-specific attributes.

## Methods

### Student-level data

This study used administrative data provided by the NYCDOE on all children enrolled in kindergarten through eighth grade in NYC public schools during the 2009–2010 school year, or approximately 584,000 individual student records (analyses using additional years of data yielded the same results/conclusions). NYC schools have conducted the *Fitnessgram* since 2005–06 as part of the district’s standards-based physical education program [18]. *Fitnessgram* requires all schools to collect measures of height and weight annually for each child, and to assess students’ aerobic fitness, muscle strength, endurance, and flexibility. All measures are taken by the school nurse or physical education teacher, following a standardized procedure. With these data, previously shown to be reliable [20], BMI was calculated and CDC height and weight charts were used along with students’ gender and age in months to identify children above the threshold for obesity and overweight. A small number of children with a biologically implausible BMI, defined by the CDC as being more than five standard deviations away from the mean, were excluded from analysis (less than 0.1% of those with non-missing values) [21]. Also excluded were students attending schools where *Fitnessgram* data were available for fewer than 60% of children in 2009–10 (only 2.2% of all K-8 student observations) and students with missing *Fitnessgram* data (3.8% of students in schools not dropped due to low *Fitnessgram* coverage). After these sample exclusions, BMI data were available for 93.3 percent of all NYC public school children in grades K-8 (N = 545,146). This study was approved by the New York University Institutional Review Board. All data are de-identified administrative data, and therefore the study was exempt from obtaining individual subject consent.

BMI and obesity indicators were matched to individual student demographics from the NYCDOE, including race/ethnicity (Black, Hispanic, White, and Asian or other race, reported in mutually exclusive categories), age at the time of the *Fitnessgram*, indicators for whether the child lives in a low-income household (measured by eligibility for the federal free or reduced price lunch program or enrollment in a universal free meals school), whether the student is foreign born, speaks a language other than English at home, or is receiving special education services. These data also include school attended and geocoded home address, the latter used to assign each child to a residential census tract. Tracts are Census Bureau designations of approximately 4,000 people—although they can range from 1,200 to 8,000 people—and are commonly used in the literature to approximate city neighborhoods. As of this writing there are roughly 2,700 tracts in NYC [22]. Using other proxies for neighborhood in our analysis, including zip code, produced similar results.

### Statistical analysis

To examine place-based variation in the prevalence of obesity among NYC public school students, we began by calculating the raw, unadjusted mean obesity rate for each neighborhood (census tract) and school. The distribution of unadjusted obesity rates is summarized, described graphically, and in the case of neighborhoods, visualized on a map of NYC census tracts. Not surprisingly, the prevalence of youth obesity varies systematically with the socioeconomic and demographic composition of neighborhoods and schools.

We then use the student-level data to adjust for differences in the observed characteristics of students across neighborhoods and schools, estimating a series of multilevel linear probability regression models (LPMs) in which an indicator for BMI above the threshold for obesity was the dichotomous outcome *y*_*is*_ (where a value of one indicates whether student *i* in neighborhood or school *s* is classified as obese), and the student characteristics shown in Table 1 are included as controls (represented in Eq (1) by the vector *X*_*is*_):

$$y_{is}=\beta_{0}+\beta^{\prime }X_{is}+\delta_{s}+u_{is}$$ (1)

To fully capture the relationship between obesity status and student demographics, we entered child age into the model as a quadratic interacted with both gender and race/ethnicity. The variation in obesity common to neighborhoods or schools that is unexplained by the student covariates *X*_*is*_ is represented by the neighborhood or school effect *δ*_*s*_. We alternately modeled *δ*_*s*_ as a random or fixed effect. In the former case, the *δ*_*s*_ were estimated as best linear unbiased predictors (BLUPs); in the latter the *δ*_*s*_ were estimated directly in the regression (effectively as a separate indicator for each neighborhood or school *s*). While the fixed effects model allows for correlation between the student predictors and unobserved characteristics of neighborhoods or schools (such as the local built environment), the variance in these estimates will tend to overstate the amount of variability in tract/school obesity rates due to sampling error. The BLUPs, in contrast, adjust for variation in group size by “shrinking” the random effect toward zero when the sample size is small. This article reports results from both approaches. In practice the correlation between the random and fixed effects was high (0.98 in the case of schools, and 0.89 for tracts).

**Table 1:** Percent overweight, and obese, overall and by student subgroup, NYC elementary and middle school students, 2009–10.

	% Underweight or Normal Weight	% Over-weight	% Obese	% of all students
All students	60.6	18.4	21.0	100.0
Characteristic:				
Male	58.4	18.4	23.2	49.8
Female	62.8	18.2	19.0	50.2
Black	60.5	18.1	21.4	27.1
Hispanic	54.3	19.9	25.8	39.6
Asian or other race	69.5	16.4	14.1	17.5
White	66.8	17.1	16.1	15.8
Low income	59.4	18.7	22.0	87.9
High income	67.7	16.5	15.7	12.1
Foreign born	65.3	18.4	16.3	12.5
English at home	61.5	17.7	20.8	57.9
Special education	56.7	18.5	24.8	11.3
N	342,351	99,216	103,575	545,146

Notes: “Low Income” is defined by eligibility for free or reduced lunch or enrollment in a universal free meal school. “Overweight” is defined as a BMI at or above the 85^th^ percentile and below the 95^th^ percentile for students of the same gender and age (in months). “Obese” is defined as a BMI at or above the 95^th^ percentile for students of the same gender and age (in months).

The estimated random (or fixed) effects were used to estimate the extent of variation in obesity across neighborhoods or schools that remains after accounting for the effects of differences in observed student characteristics across places. The between-neighborhood and between-school standard deviations are reported, along with adjusted obesity rates at various percentiles of the random (or fixed) effects distribution, including the 1^st^ (least obese), 5^th^, 25^th^, 50^th^, 75^th^, 95^th^, and 99^th^ (most obese) percentiles. For the adjusted obesity rates, we add the predicted obesity rate for the tract (or school), net of any tract (or school) effect and fixing the characteristics of its student population at the city averages, and the estimated tract- (or school-) specific random (or fixed) effect. Variation in the adjusted obesity rates comes entirely from the random (or fixed) effects, and represents unobserved place-specific factors which could potentially be influenced by public policy, such as the food or built environment. For comparison, percentiles of the unadjusted obesity rate distribution are also shown.

In our concluding section, we use the estimated neighborhood and school effects to simulate how the overall obesity rate among NYC elementary and middle school children would change if—holding all else equal—students attending higher obesity schools or living in higher obesity neighborhoods had the benefit of attending a lower obesity school or living in a lower obesity neighborhood, applying several possible targets for neighborhood or school effects.

To perform these simulations, we assigned each student in our analytic sample a predicted probability of having a BMI above the obesity threshold ($\hat{y_{is}}$), based upon the estimated regression coefficients from equation (2) and their actual characteristics (gender, race/ethnicity, low income and immigrant status, etc.). However, rather than using the student’s *actual* neighborhood or school effect in the prediction ($\hat{\delta_{s}}$), we assign a target neighborhood or school effect (δ_*T*_*)* to students with estimated neighborhood or school effects above this target. We then calculate a new “simulated” citywide obesity rate based on these predicted values for all students.

In these simulations, we experiment with four target neighborhood or school effects. In the first case, the *median scenario*, we assign students in schools (neighborhoods) with a $\hat{\delta_{s}}$ *above* the median to the median $\hat{\delta_{s}}$ Cases two through four repeat this exercise, but with more aggressive targets for reducing place effects on obesity. These assign students in schools (neighborhoods) with a $\hat{\delta_{s}}$ above the 25^th^, 10^th^, and 5^th^ percentiles of the distribution to these target levels *δ*_*T*_ We refer to these as the 25^th^, 10^th^ and 5^th^ percentile scenarios, respectively. Finally, a comparable simulation is performed using target neighborhood *and* school effects simultaneously. In this case students above a given threshold for *either* neighborhood or school effects are assigned the random effect for that target level.

Put simply, each of the simulations addresses the following question: given the observed variation across neighborhoods and schools in obesity among observationally similar students, by how much would the citywide obesity rate decline if students living (or attending school) in higher obesity neighborhoods (or schools) instead lived (or attended school) in a lower obesity neighborhood (or school)? The magnitude of this difference will be suggestive of the extent to which place-based interventions could reduce child obesity.

## Results

### Descriptive statistics

Descriptive Statistics for the analytic sample of students are reported in the top row and rightmost column of Table 1. Twenty one percent of all students in the sample were classified as obese, and an additional 18.4 percent were classified as overweight but not obese. Roughly 27 percent of the sample is Black, 40 percent is Hispanic, 18 percent Asian, and 16 percent white. About 13 percent are foreign born and 58 percent primarily speak English at home. Not surprisingly, there were insignificant differences between the analytic sample and the full population of NYC public elementary and middle school students, since our sample represents more than 93 percent of all public school students in grades K-8.

The three leftmost columns in Table 1 report the prevalence of obesity and overweight for select student subgroups, with separate results reported by gender, race/ethnicity, and low income, immigration, and special education status. Obesity and overweight among children in NYC public schools varies systematically with sociodemographic and other background characteristics, a pattern consistent with the existing literature [23]. In NYC, male, Hispanic, black, and low income children are more likely to be obese than female, white, Asian, and foreign born children. We calculated the same descriptive statistics for other years of data available to us (2007–08 and 2008–09) and the results were comparable. This paper focuses on one year of data, for ease of exposition.

### Unadjusted neighborhood- and school-level variation in obesity

Columns (1) and (4) of Table 2 show how the prevalence of obesity among NYC public school children varies across neighborhoods (census tracts) and schools, prior to adjusting for differences in observed student characteristics. As a summary measure of this variability, the first row reports the standard deviation (s.d.) in neighborhood or school-level obesity rates. Prior to adjusting for student characteristics, the s.d. in obesity rates across neighborhoods and schools were 6.0 and 6.4 percentage points, respectively. This translates into a roughly 12 to 13 percentage point difference in obesity rates between neighborhoods (or schools) one s.d below and one s.d. above the NYC average, compared to a mean obesity rate of 21 percent.

**Table 2:** Variation in obesity rates across neighborhoods (census tracts) and schools before and after adjustment for student characteristics, NYC public elementary and middle school students, 2009–10.

	Neighborhoods			Schools		
	(1)	(2)	(3)	(4)	(5)	(6)
	Unadj. rates	Adjusted (RE)	Adjusted (FE)	Unadj. rates	Adjusted (RE)	Adjusted (FE)
SD(*δ*_*s*_)	6.0	2.6	4.8	6.4	4.3	5.4
Percentile:						
1^st^	4.2%	16.7%	7.2%	4.2%	11.0%	7.7%
5^th^	8.4%	17.9%	11.5%	10.6%	14.2%	12.2%
25^th^	16.4%	19.6%	17.8%	17.9%	18.9%	18.2%
Median	20.4%	20.7%	20.5%	21.9%	21.4%	21.6%
75^th^	23.8%	21.9%	23.4%	25.6%	23.6%	24.2%
95^th^	28.5%	23.9%	27.7%	31.1%	27.3%	29.5%
99^th^	32.8%	25.5%	31.7%	36.0%	31.6%	34.5%
Mean	19.8%	20.8%	20.3%	21.5%	21.2%	21.2%
N	2,084	2,084	2,084	1,077	1,077	1,077

*Notes*: For each tract (or school) the “adjusted” rate is calculated as the sum of: (1) the predicted citywide obesity rate, net of the tract (or school) effect; and (2) the tract- (or school-) specific random (or fixed) effect. The first (“adjusted”) part is the same in all tracts (schools), while the second part varies by tract (school). Thus, the variation in the “adjusted” rates is due entirely to the tract or school effects. Predicted obesity rates come from a multilevel linear probability model with controls for a three-way interaction between race, gender, and age, as well as grade level, immigration status, home language, eligibility for free or reduced price lunch, and participation in special education services.

**Fig 1:**
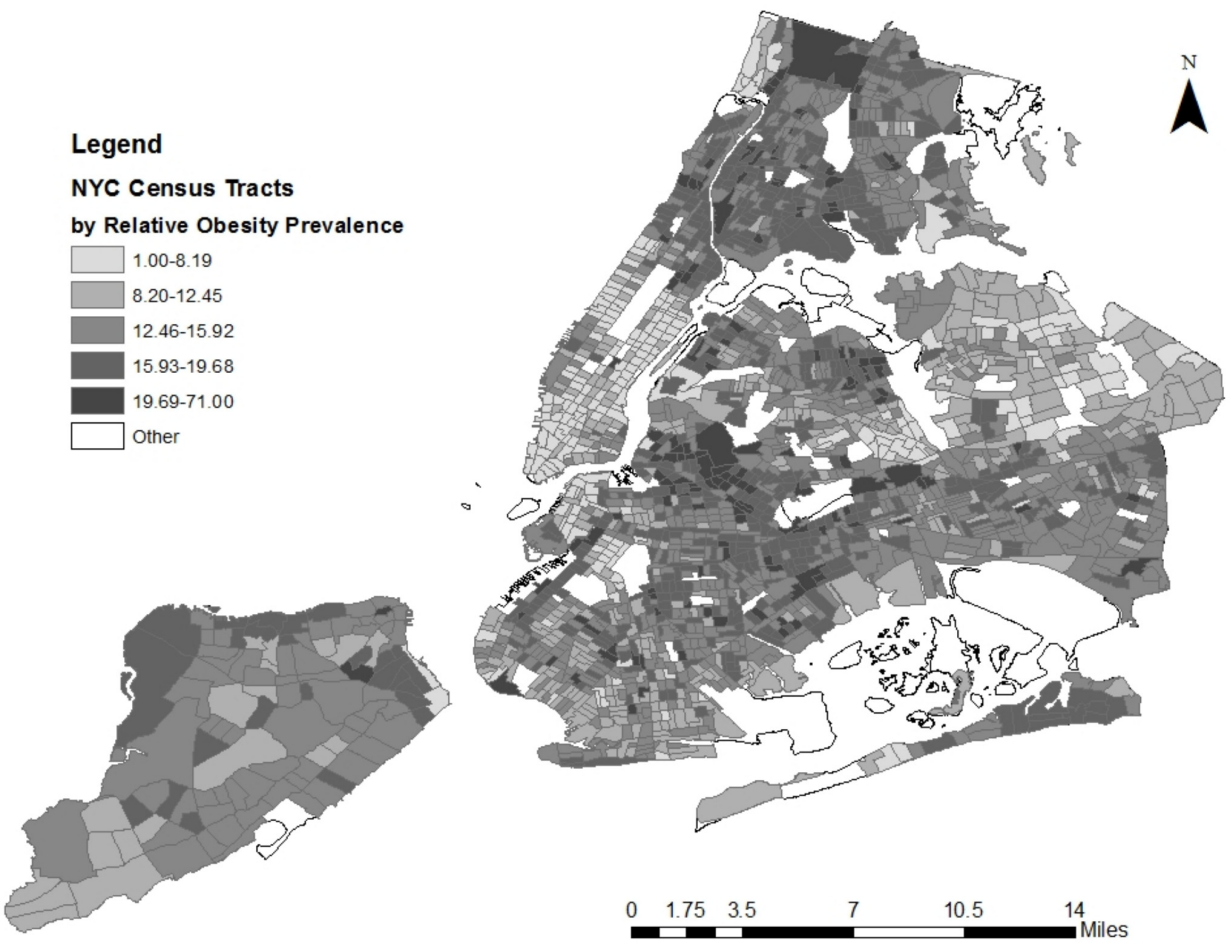
Relative obesity prevalence among NYC schoolchildren, by census tract, 2009–10.

Another way of characterizing this variability is by comparing obesity rates at specific percentiles of the distribution of neighborhoods or schools; these percentiles are reported in the other rows of Table 2, columns (1) and (4). Prior to adjusting for student characteristics, the least obese neighborhoods, at the 1^st^ percentile, had an unadjusted obesity rate of only 4.2 percent. The neighborhood at the 25^th^ percentile had triple this rate, at 16.4 percent, while the neighborhoods at the 75^th^ and 99^th^ percentiles had unadjusted obesity rates of 23.8 and 32.8 percent, respectively. We observe similar differences across percentiles of the school obesity rate distribution (column (4)). Fig 1 shows how the prevalence of obesity among NYC public schoolchildren varies spatially across neighborhoods (census tracts). Following Black et al. (2010) [19], for Fig 1 we assigned tracts with the lowest obesity rates among NYC schoolchildren a prevalence ratio of 1.0. (The tracts with the lowest obesity rates were DUMBO in Brooklyn and Greenwich Village in Manhattan, with roughly 1 percent of students classified as obese). The prevalence ratio in other tracts is relative to this baseline, such that a tract with a prevalence ratio of 10, for example, has an obesity rate ten times that of the lowest obesity tract.

After calculating individual-level obesity status using administrative data on height, weight, gender and age, we assigned each individual to a residential census tract in order to calculate obesity prevalence by census tract. We assigned the census tract with the lowest obesity rate a prevalence ratio of 1.0, and the prevalence ratio in other tracts is relative to this baseline.

In a majority of tracts, the youth obesity rate exceeds 20 percent, but there is significant geographic variation across the city. Neighborhoods in Harlem and Washington Heights in Manhattan, the South and North Bronx, and East Brooklyn, for example, have substantially higher obesity rates than neighborhoods on the Upper East and Upper West Sides of Manhattan, and in the eastern sections of Queens. Perhaps not surprisingly, tracts with proportionately higher obesity rates also tend to be those with higher concentrations of poverty, black and Hispanic residents, and native born residents. In the next section we examine whether this variation can be explained by accounting for observed characteristics of students that tend to be associated with BMI.

### Regression-adjusted neighborhood- and school-level variation in obesity

Columns (2), (3), (5), and (6) of Table 2 report the s.d. in neighborhood and school effects after adjusting for observed student characteristics. Columns (3) and (6) report results from the random effects regression models, while (2) and (5) are based on the fixed effects models. (Estimated coefficients for the other explanatory variables are available from the authors upon request). As expected, variability in the prevalence of obesity is reduced when adjusting for these characteristics, but it remains substantial. Based on the random effects model, the s.d. in adjusted neighborhood and school effects were 2.6 and 4.3 percentage points respectively. This corresponds to a 5.2 percentage point difference in obesity between neighborhoods one s.d. below and one s.d. above the city average, and an 8.6 percentage point difference between schools one s.d. below and one s.d. above the city average. On a baseline average neighborhood (school) obesity rate of 19.8 (21.5) percent, these adjusted differences remain significant in size.

Variability across neighborhoods and schools in adjusted obesity rates can also be characterized by comparing specific percentiles of the distribution. After adjusting for observed student characteristics (for example, using the fixed effects model), the least obese neighborhoods, at the 1^st^ percentile, had an obesity rate of 7.2 percent. The neighborhood at the 25^th^ percentile had more than double this, at 17.8 percent, while the neighborhoods at the 75^th^ and 99^th^ percentiles had adjusted rates of 23.4 and 31.7 percentage points, respectively. Regression adjustment for student characteristics reduces the differences between percentiles of the neighborhood and school obesity rate distribution, but significant variation remains.

### Simulating the impact of reducing neighborhood or school effects on obesity

**Fig 2:**
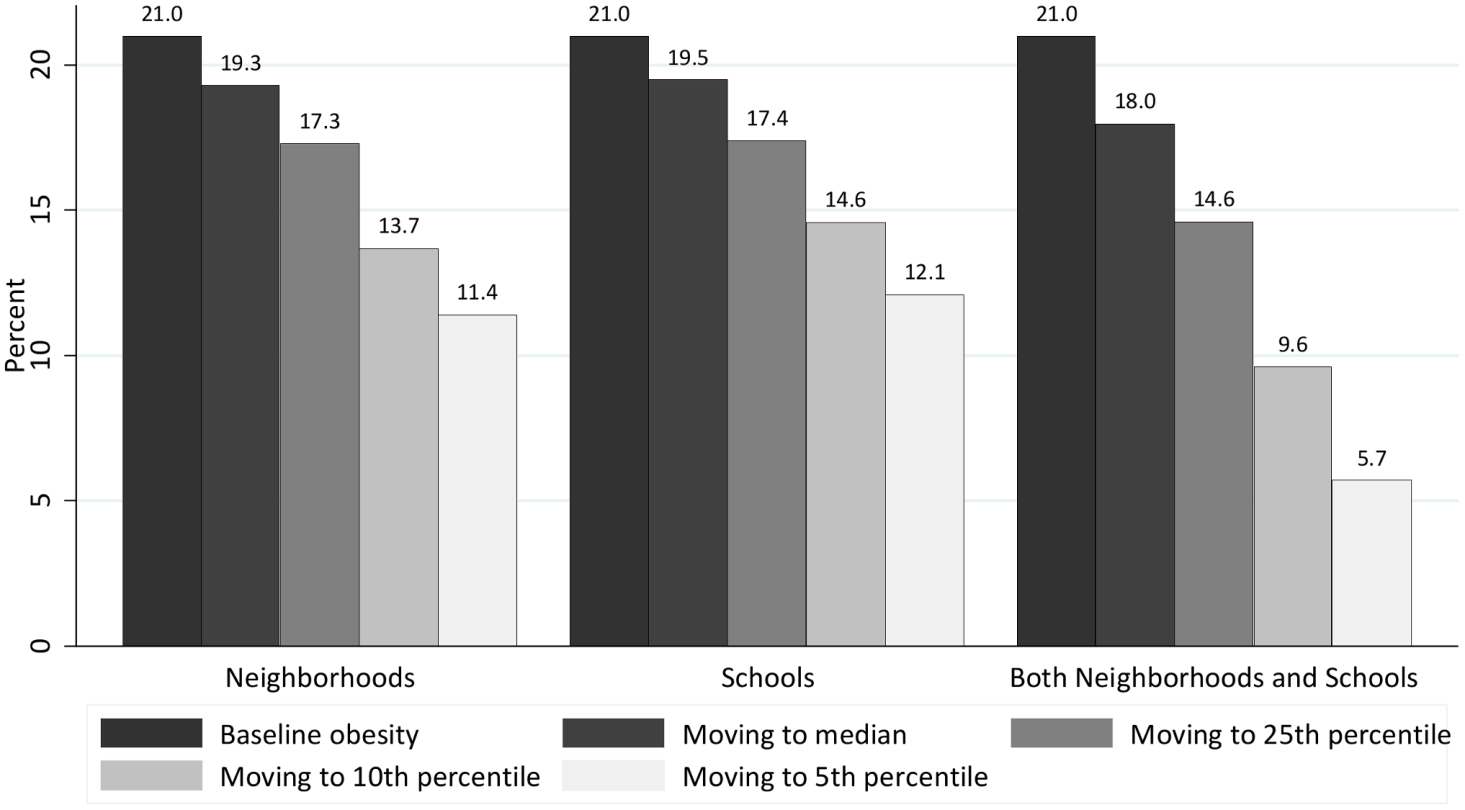
Simulated citywide obesity rates from moving students to “low-obesity” neighborhoods and schools, NYC public elementary and middle school students, 2009–10.

Finally, we used our regression results and estimated neighborhood and school effects to simulate how the overall obesity rate among NYC elementary and middle school children would change if—holding all else equal—students attending higher obesity schools or living in higher obesity neighborhoods attended a lower obesity school or lived in a lower obesity neighborhood. Results from these simulations are shown in Fig 2.

For these simulations, each student is assigned a predicted probability of being obese, given their observed characteristics. Their estimated tract (or school) effect is then added to this predicted probability. In the median scenario, the student’s actual tract (school) effect is replaced by the median tract (school) effect, if their own effect exceeds the median. We do the same for tract (and school) effects above the 25^th^, 10^th^, and 5^th^ percentiles. The “both” simulation is based on a regression model with census tracts and schools in the same model. All models include the following student-level predictors: a three-way interaction between race, gender, and age, as well as grade level, immigration status, home language, eligibility for free or reduced price lunch, and participation in special education services.

From a baseline citywide obesity rate of 21 percent, the *median scenario*—in which students in neighborhoods above the median in the random effects distribution are assigned the random effect from the median neighborhood—reduces the overall obesity rate by roughly 1.7 percentage points to 19.3 percent (an 8.1 percent decline). Performing the same exercise for *schools*—that is, the median scenario for schools, rather than neighborhoods—reduces the obesity rate to 19.5 percent, a comparable decline. If students in higher obesity neighborhoods *or* higher obesity schools were assigned the effect at the median, the citywide obesity rate would fall to 18.0 percent, a 2.7 percentage point (or 14.3 percent) decrease.

Using more aggressive targets, if students in neighborhoods above the 25^th^ percentile were assigned the neighborhood effect at that point in the distribution, the decline in the overall obesity rate would be 3.7 percentage points, to 17.3 percent. Using the 10^th^ percentile as a target the decline in the overall obesity would be 7.4 percentage points, to 13.7 percent. Using the 25^th^ and 10^th^ percentiles for *schools* would yield similar results. It is worth noting that even the median targets are ambitious; for example, under the median scenario, the average tract above the median experiences a 3.4 percentage point reduction in obesity under the simulation.

## Discussion

These simulations show that reducing the place-based effect of obesity of students living (or attending school) in high obesity neighborhoods (or schools) to the level of observationally similar students living in (or attending school) in lower obesity neighborhoods (or schools) would have a meaningful if modest effect on the overall rate of obesity in NYC. If policies were able to reduce neighborhood (or school) effects to that of the median tract (school), we estimate a 1.5 to 1.7 point (or 8–12 percent) reduction in the obesity rate. More aggressive targets would yield greater reductions. There are two important caveats to keep in mind when interpreting these results, however. First, as noted above, even the median scenario is a rather ambitious target, requiring a 3–4 percentage point reduction in obesity among the highest obesity tracts (or schools). Second, students in NYC are unevenly distributed across neighborhoods and schools. A larger share of NYC students live (or attend school) in higher obesity neighborhoods (or schools) than in lower obesity neighborhoods (or schools). On the one hand, this means targeted, place-based interventions may be an efficient approach, since changing a high obesity neighborhood affects a larger number of students than changing a lower obesity neighborhood. On the other hand, lower obesity neighborhood (or school) effects may be particularly unrealistic targets, since they reflect a relatively smaller share of the population.

Using a large, representative administrative dataset of public school children, this study explored the empirical “case” for place-based policy interventions to reduce child obesity in a large urban area. We began by measuring the extent to which obesity varies across neighborhoods and schools in NYC without adjusting for differences in observed student characteristics. This analysis revealed significant differences across places, with (for example) a 7.4 percentage point difference in the prevalence of obesity between neighborhoods at the 25^th^ and 75^th^ percentiles, and an 8.6 point difference between the 25^th^ and 75^th^ percentiles of schools. Controlling for differences in student characteristics that tend to be associated with obesity reduces the variability across neighborhoods and schools, but leaves a considerable amount “unexplained.” That is, even when adjusting for differences in sociodemographic composition, we observe systematic differences across neighborhoods and schools that suggest a potential for place-based interventions to reduce child obesity. Another important observation is that low obesity neighborhoods and schools contain, on average, a disproportionately smaller share of students relative to high-obesity neighborhoods. Thus, targeting interventions at high obesity places effectively reaches a larger proportion of obese students. Unfortunately, the relative scarcity of low obesity neighborhoods (schools) in NYC means that even if the most obese neighborhoods and schools were to substantially improve their obesity rates toward the median or lower, population obesity rates would change modestly.

These results present differences across neighborhoods and schools in a somewhat different light than previous research. Much of the work focusing on neighborhoods examines specific neighborhood-level factors that are associated with differences in obesity rates at the individual or neighborhood level [8, 19, 24–27]. In contrast, we focused on the overall potential for place-based interventions which reduce the magnitude of neighborhood and school effects to lower the citywide prevalence of obesity. A particularly important innovation was the examination of variability across schools. With few exceptions [28] the literature on school-level obesity rates is largely focused on the effects of individual programs or policies [29–31]. This study’s focus, on the other hand, provides important bounds on the potential influence of approaches that target schools.

Neighborhoods and schools clearly matter, but their overall influence on obesity rates may well be more limited than hoped. Even if one could identify the characteristics of these neighborhoods and schools that are causally related to lower obesity (a large assumption), and successfully identify ways to change them and make those changes happen, the overall population-wide effect would be modest. These modest effects could arise for two reasons. First, it could be that contextual factors alone have a limited influence on obesity. Second, it could be that even “out-performing” neighborhoods and schools need further improvement to fully recognize their potential in bettering childhood obesity. To be sure, even the modest reductions in childhood obesity simulated here would be welcome. The results suggest that differences related to school food, the built environment and the food environment could have meaningful effects. That said, these results also suggest that other approaches need to be considered along with neighborhood and school-level interventions and/or a deepening of current efforts on this front.

This work is not without limitations. The *Fitnessgram* relied on each school to collect the height and weight data necessary to calculate BMI, although this procedure is standardized, and our large sample size should limit the influence of noise in the estimates. Given the macro-level perspective taken by this study, the researchers did not observe or measure specific contextual factors, policies, or programs that potentially explain variation across neighborhoods and schools. The investigation of specific mechanisms is left to future research. This study also focused on obesity using BMI and not alternative measures of childhood weight status (although BMI is the most commonly used of these measures). BMI is a measure with inherent limitations, and may particularly understate obesity in some groups (notably Asians) [1]. The data include a usually rich, but still limited, set of individual student controls. They could omit some potentially important home and parental factors that explain the systematic association between neighborhoods (and schools) and the prevalence of obesity. These predictors could reduce the estimated magnitude of school and neighborhood effects. We also only examine variation across locations at a single point in time, and not over time. This could have led to a different set of conclusions. Finally, while census tracts are a reasonable analog to NYC neighborhoods, they may not necessarily reflect the sphere in which residents spend most of their time (though they are probably more accurate for children than for working/commuting adults).
